# Efficacy and safety of endoscopic procedures for common bile duct stones in patients aged 85 years or older: A retrospective study

**DOI:** 10.1371/journal.pone.0190665

**Published:** 2018-01-03

**Authors:** Tomoya Iida, Hiroyuki Kaneto, Kohei Wagatsuma, Hajime Sasaki, Yumiko Naganawa, Suguru Nakagaki, Shuji Satoh, Haruo Shimizu, Hiroshi Nakase

**Affiliations:** 1 Department of Gastroenterology, Muroran City General Hospital, Muroran, Hokkaido, Japan; 2 Department of Gastroenterology and Hepatology, Sapporo Medical University School of Medicine, Sapporo, Hokkaido, Japan; Sapienza University of Rome, S. Andrea Hopital, ITALY

## Abstract

The Endoscopic procedures for common bile duct (CBD) stones are reportedly safe in the elderly patients. However, the definition of the elderly is different in each report. If the elderly are defined as people aged 85 years or older, data on the effectiveness and safety of endoscopic retrograde cholangiopancreatography (ERCP) for CBD stones are limited. This study investigated the efficacy and safety of endoscopic procedures for CBD stones in patients aged 85 years or older. 1,016 consecutive ERCP procedures were performed at our institution from January 2009 to December 2014. Of these, 235 cases with CBD stones were finally analyzed. Group A patients were younger than 85 years and Group B patients were 85 years or older. Patient background, details of endoscopic therapy, complications, and related factors were retrospectively reviewed for 185 cases in Group A, and 50 cases in Group B. Patients in Group B showed high rates of dementia and cerebrovascular disorders and larger CBD stones and diameters, in comparison with patients in Group A. The complete removal rate of bile duct stones was slightly higher in Group A. However, there was no difference between the two groups in recurrence rate of CBD stones, complication and mortality rates, and length and cost of hospitalization. Despite some differences between the two groups, endoscopic procedures for CBD stones in patients aged 85 years or older can be performed effectively and safely without increasing medical costs.

## Introduction

Life expectancy is increasing worldwide, particularly in Japan. The increasing proportion of elderly people in the population is accompanied by an increase in the prevalence of bile duct stones [1,2]. In determining a therapeutic strategy for these patients, safety and therapeutic effectiveness are important because the elderly often have chronic diseases that may be exacerbated as procedure-related adverse events.

The Tokyo Guidelines for the diagnosis, classification, and treatment of acute cholangitis were published in 2007 and updated in 2013 [3,4]. Although treatment is determined based on disease severity in the Tokyo Guidelines, endoscopic treatment has an important role in all degrees of severity.

Endoscopic treatment for common bile duct (CBD) stones is evolving. It is less invasive than surgery [2], and is reportedly safe in elderly patients [5–7]. However, the definition of the elderly was different in each report. If the elderly are defined as people aged 85 years or older, data on the effectiveness and safety of endoscopic retrograde cholangiopancreatography (ERCP) for CBD stones would be limited [7]. Furthermore, no reports have evaluated the operator skills or medical costs associated with treatment of CBD stones in the elderly. This study investigated the efficacy and safety of endoscopic procedures for CBD stones in patients aged 85 years or older.

## Materials and methods

### Patients

Between January 2009 and December 2014, 1,016 consecutive ERCP procedures were performed at our institution. The patients who were hospitalized for CBD stones and received endoscopic therapy within this period were included in this study. We excluded patients with a diagnosis of CBD stone during hospitalization for other diseases, those who had a history of ampullary treatment, and those who had never received therapeutic endoscopy because of spontaneous stone passage or underwent surgery. In addition, we also excluded patients who have not been followed more than 3 months and those who have never been undergone imaging examination such as abdominal ultrasonography (US), computed tomography (CT), or magnetic resonance cholangiopancreatography (MRCP) in follow-up period to monitor for a recurrence of CBD stone.

**Fig 1:**
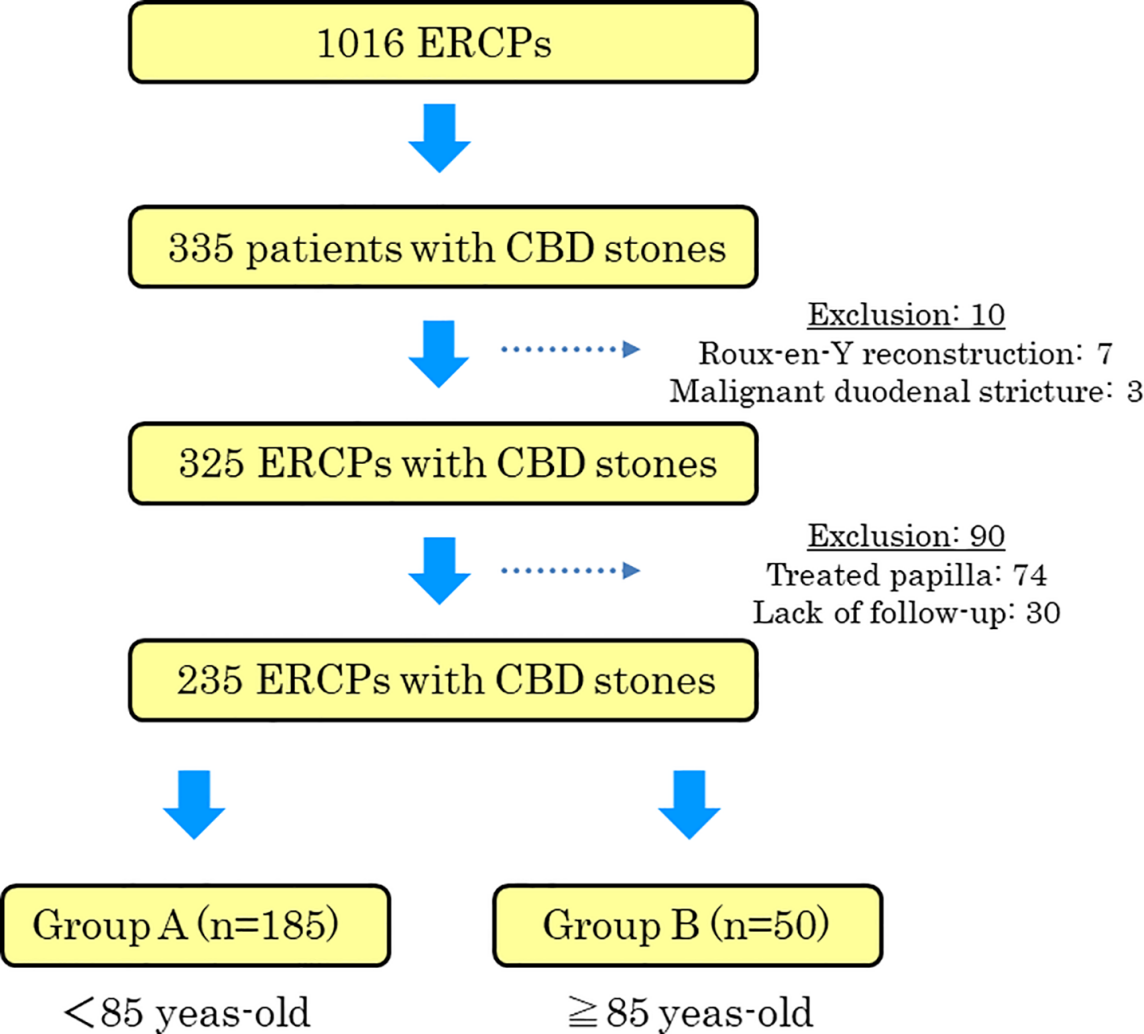
Schematic diagram of the patients enrollment in this study.

During this period, 325 of 335 patients had endoscopic treatment for CBD stones. Seven of the remaining 10 patients had undergone Roux-en-Y reconstruction and could not be treated endoscopically. Three patients with malignant duodenal strictures due to pancreatic cancer could not be treated because the endoscope could not pass through the second portion of the duodenum. In addition, 90 patients were excluded because of having history of papilla treatment and/or lack of follow-up. Group A patients were younger than 85 years and Group B patients were 85 years or older. Finally, Group A was composed of 185 patients, and Group B, 50 patients (Fig 1).

### Ethical considerations

This study was approved by the institutional review board committee of Muroran City General Hospital (No. 2016–7). The data used in this study were de-identified, and released to the public for research purposes. Therefore, the institutional review board of Muroran City General Hospital waived informed consent from the enrolled patients. This study was performed in accordance with the Declaration of Helsinki.

### Procedures

ERCP was performed by an expert operator with experience in more than 3,000 cases, and by trainees with an average experience of 30 cases, under supervision by the expert. Physicians who had been mainly in charge of patients started the endoscopic procedure regardless of difficulty level. Trainees started the procedure and switched with the expert physician when they were unable to achieve front-viewing of the papilla within 5 minutes, cannulation within 15 minutes, and completion within 60 minutes from 30 minutes after starting the procedure.

Pentazocine (PZ) and midazolam (MDZ) were administered intravenously for conscious sedation, with monitoring of peripheral oxygen saturation and blood pressure during the procedure. The initial doses of PZ and MDZ were 15 mg and 1.25–2.5 mg, respectively, and were supplemented according to the response. Oxygen supplementation through a nasal cannula was used as necessary. Vital signs (blood pressure, heart rate, and arterial oxygen saturation), patient responses, and sedative doses were recorded by a nurse during the procedure.

Standard duodenoscopes (JF-260V, TJF-240, Olympus Medical Systems Co.) were used for the procedures. We also used 2T-2Q260M, CF-Q240AI, and PCF-Q260AL/I endoscopes (Olympus Medical Systems Co.) in patients who had undergone gastric surgeries such as Billroth I and Billroth II.

Wire-guided cannulation was used for bile duct catheter insertion [8]. When a biliary approach using a standard method failed, a precut rescue technique with a needle knife (RX Needle knife XL; Boston Scientific Co.) was performed. Endoscopic sphincterotomy (EST) using sphincterotomes (CleverCut2V; Olympus Medical Systems Co.), endoscopic papillary balloon dilation (EPBD) using a balloon dilation catheter with a diameter of 8 or 10 mm (Hurricane RX; Boston Scientific Co.), or endoscopic papillary large balloon dilation (EPLBD) using a large dilating balloon (GIGA; Century Medical Inc.) was performed to remove stones in most cases. While EST (basic medium incision) was performed for the initial papillotomy, EPBD was an option, depending on the use of antithrombotic drugs or the presence of periampullary diverticulum. EPLBD was selected for patients with three or more stones and adequate dilation from the lower to the hilar bile duct, even if stones were 12 mm or longer or 10 mm in diameter. For stone removal, standard techniques (basket, balloon catheter, or mechanical lithotripter) were used. A mechanical lithotripter was used to crush stones too large to remove. Extracorporeal shock wave lithotripsy was performed for large stones that were difficult to crush with a mechanical lithotripter. In general, complete CBD stone removal was attempted at the time of each procedure. Complete stone removal was confirmed direct cholangiography and intraductal ultrasonography (IDUS). A 7.0Fr endoscopic nasobiliary drainage (ENBD) tube (CLINY ENBD tube; Create Medic Co.) was placed depending on the judgment of the operator. When complete stone removal was not achieved, a 7.0Fr ENBD tube or a 7.0Fr straight or double-pigtail stent (Flexima Biliary Stent System; Boston Scientific Co.) was placed for bile duct drainage. Stent exchange was performed only when acute cholangitis recurred. In addition, we placed a 5Fr×3cm naturally dropping off prophylactic pancreatic stent (Geenen Pancreatic stent; Cook Medical Co.) when we performed pancreatic guidewire cannulation.

After the procedure, we administered 0.2–0.5 mg of flumazenil. As a preventive measure against pancreatitis after ERCP, sufficient rehydration was given, in addition to protease inhibitors or ulinastatin in all the cases. In particular, 100mg of gabexate mesylate or 150,000 units of ulinastatin was dissolved in 500ml of 5% dextrose solution and administered via intravenous infusion immediately after the ERCP procedures and the next morning. Furthermore, immediately after the ERCP procedures, the patients received 25 to 50mg diclofenac suppositories depending on the judgment of the operator.

For the patients who received placement of an ENBD tube, the presence of the remaining stones was checked under fluoroscopy on the day after treatment. For patients with remaining stones, endoscopic treatment was performed again considering their general condition. As follow-up after the treatment, physical examination, blood tests, abdominal US, CT, or MRCP, were performed to monitor for any recurrence of CBD stone. For the patients with suspected recurrence of CBD stone, ERCP was performed as much as possible. For patients who had a recurrence of CBD stone, endoscopic removal of the stones was performed.

### Outcome

The primary endpoint was defined as the complete stone removal rate and the incidence of complications in Group A and Group B in this study. In addition, we also determined the recurrence rate of CBD stone as well as the length of hospitalization and the cost of hospitalization for the purpose of examination of medical cost in both groups.

### Data extraction

The following information was obtained from medical charts and imaging studies: the proportion of patients who underwent endoscopy, patient background (age, sex, Eastern Cooperative Oncology Group performance status score, presence of concomitant diseases, use of antithrombotic drugs or ursodeoxycholic acid, presence of gallbladder in situ or periampullary diverticulum or reconstructed gastrointestinal tract or cholangitis at diagnosis), details of the endoscopic treatment (cases with symptomatic, procedure time, the number of procedure, dose of MDZ, diameter of CBD, the number and diameter of stones, ampullary treatments, cases with complete bile duct stone removal, cases with pancreatic stent placement, use of prophylactic NSAIDs, cases with procedure initiation and completion by trainees), complications, factors associated with complications, length of hospitalization, cost of hospitalization, mortality, cholecystectomy rate after endoscopic procedure, recurrence rate, observational period, and factors related to non-recurrence.

The number and diameter of stones were determined based on the basis of the combined findings of CT, MRCP, and ERCP. The number of sludge or muddy stone was counted as 0 because of the difficulty of counting them as stones. The diameter of stones was defined as the long diameter of the largest stone. Recurrence was defined as the presence of stones after complete removal during the follow up described above. Complications were classified as intra-procedural or post-procedural. Cardiorespiratory suppression was defined as peripheral oxygen saturation < 90% and/or systolic blood pressure < 90 mmHg at any time during the procedure. The occurrence and severity of post-ERCP pancreatitis, cholangitis, and bleeding were defined in accordance with the 1991 consensus guidelines by Cotton et al. [9]. The cost of hospitalization was calculated as the number of health insurance reimbursement points, based on the combined diagnosis and procedure. Patients who underwent surgery or treatment for other disorders were excluded from the analysis.

### Statistical analysis

Data were analyzed using StatView version 5.0. (SAS Institute Inc.). Results were reported as mean and standard deviation for variables with a normal distribution, and as median, minimum, and maximum values for variables with a non-normal distribution. The significant differences in the variable characteristics were investigated using Fisher’s exact test for categorical variables and a Student’s t-test for continuous variables. The multivariable logistic regression analysis was used for factors associated with procedure-related complications and recurrences. Two-sided hypothesis testing was performed, with a p-value of ≦0.05 considered statistically significant.

## Results

The patients’ characteristics are shown in Table 1. The mean age was 71±12.8 years in Group A and 88±3.0 years in Group B. In Group A, the proportion of men was significantly greater than that in Group B (63.2% [117/185] vs. 40.0% [20/50], p = 0.004). Significant differences were also observed in median performance status score (1 vs. 2, p<0.001) and in the proportion of patients with gallbladder in situ (79.5% [147/185] vs. 64.0% [32/50], p = 0.038) between Group A and B. However, no significant differences in comorbidities were found between the groups, except for the higher rate of cerebrovascular disorders and dementia in Group B. In addition, no significant differences were found between the groups with regard to the presence or absence of cardiac disorders, periampullary diverticulum, reconstructed gastrointestinal tract, cholangitis at diagnosis, severity of cholangitis, or the use of antithrombotic drugs or ursodeoxycholic acid.

**Table 1:** Patients’ characteristics and details of the endoscopic procedures.

	Group A (< 85y)	Group B (≧ 85y)	p value
	(n = 185)	(n = 50)	
Patient characteristics			
Age (mean±SD) (years)	71±12.8	88±3.0	
Male (N, %)	117 (63.2)	20 (40.0)	0.004
PS (median, range)	1 (0–4)	2 (0–4)	<0.001
Concomitant diseases (N, %)	168 (90.8)	50 (100)	0.027
Cerebrovascular disorder (N, %)	40 (21.6)	20 (40.0)	0.011
Dementia (N, %)	5 (2.7)	9 (18.0)	<0.001
Cardiac disorder (N, %)	26 (14.1)	8 (16.0)	NS
Anti-thrombotic drug (N, %)	63 (34.1)	18 (36.0)	NS
Ursodeoxycholic acid (N, %)	37 (20.0)	10 (20.0)	NS
Gallbladder in situ (N, %)	147 (79.5)	32 (64.0)	0.038
Periampullary diverticulum (N, %)	78 (42.2)	26 (52.0)	NS
Reconstructed gastrointestinal tract (N, %)	11 (5.9)	4 (8.0)	NS
Cholangitis at diagnosis (N, %)	123 (66.5)	36 (72.0)	NS
Severity of cholangitis: Mild (N, %)	74 (40.0)	19 (38.0)	NS
: Medium (N, %)	32 (17.3)	10 (20.0)	NS
: Severe (N, %)	17 (9.2)	7 (14.0)	NS
Detail of endoscopic procedure			
For symptomatic (N, %)	138 (74.6)	39 (78.0)	NS
Procedure time (mean±SD) (min)	42.0±7.4	45.2±7.6	NS
Number of procedures (mean±SD)	1.1±0.2	1.3±0.3	NS
Dose of MDZ (mean±SD) (mg)	6.3±1.4	5.2±1.6	0.01
Diameter of CBD (mean±SD) (mm)	9.2±1.6	11.2±1.8	<0.001
Diameter of stones (mean±SD) (mm)	7.0±0.9	8.2±1.0	0.01
Number of stones (median, range)	1 (0–12)	2 (0–8)	NS
Number of cases with muddy stone/sludge (N, %)	10 (5.4)	4 (8.0)	NS
EST (N, %)	150 (81.1)	38 (76.0)	NS
EPBD (N, %)	35 (18.9)	9 (18.0)	NS
EPLBD (N, %)	25 (13.5)	8 (16.0)	NS
ENBD (N, %)	165 (89.2)	40 (80.0)	NS
Complete removal for stones with ENBD (N, %)	164 (88.6)	37 (74.0)	0.013
Complete removal for stones without ENBD (N, %)	20 (10.8)	10 (20.0)	NS
Complete removal for stones (N, %)	184 (99.5)	47 (94.0)	0.031
Pancreatic stent placement (N, %)	35 (18.9)	11 (22.0)	NS
Prophylactic NSAIDs use (N, %)	13 (7.0)	3 (6.0)	NS
Starting procedure by trainee (N, %)	150 (81.1)	36 (72.0)	NS
Complete removal for stones by trainee (N, %)	126 (68.1)	35 (70.0)	NS
			(Fisher’s exact test, Student’s t-test)

SD: standard deviation, PS: performance status, NS: not significant, MDZ: midazolam, CBD: common bile duct, EST: endoscopic sphincterotomy, EBPD: endoscopic papillary balloon dilation, EPLBD: endoscopic papillary large balloon dilation, ENBD: endoscopic naso-biliary drainage

Details of the endoscopic procedures are also shown in Table 1. The respective significantly different results between Group A and Group B were as follows: dose of MDZ, 6.3±1.4 mg vs. 5.2±1.6 mg, p = 0.01; diameter of CBD, 9.2±1.6 mm vs. 11.2±1.8 mm, p<0.001; diameter of stones, 7.0±0.9 mm vs. 8.2±1.0 mm, p = 0.01; rate of complete removal for stones with ENBD, 88.6% (164/185) vs. 74.0% (37/50), p = 0.013; rate of complete removal for stones, 99.5% (184/185) vs. 94.0% (47/50), p = 0.031.

No significant differences were found between the groups in terms of the incidence of complications during endoscopic procedures (8.6% [16/185] vs. 14.0% [7/50]), decreased blood pressure (1.6% [3/185] vs. 0% [0/50]) or peripheral oxygen saturation level (2.7% [5/185] vs. 2.0% [1/50]). No significant differences were found between the groups in terms of the incidence of complication after endoscopic procedures, including pancreatitis (16.8% [31/185] vs. 12.0% [6/50]), cholangitis (3.2% [6/185] vs. 2.0% [1/50]), and bleeding (1.1% [2/185] vs. 4.0% [2/50]) (Table 2).

**Table 2:** Complications during or after the endoscopic procedures.

Complications during the	Group A (< 85y)	Group B (≧ 85y)	p value
endoscopic procedure	(n = 185)	(n = 50)	
Overall (N, %)	16 (8.6)	7 (14.0)	NS
Blood pressure reductions (N, %)	3 (1.6)	0 (0)	NS
Peripheral oxygen			
saturation reductions (N, %)	5 (2.7)	1 (2.0)	NS
Complications after the	Group A (< 85y)	Group B (≧ 85y)	p value
endoscopic procedure	(n = 185)	(n = 50)	
Overall (N, %)	37 (20.0)	9 (18.0)	NS
Pancreatitis (N, %)	31 (16.8)	6 (12.0)	NS
(Mild/Medium/Severe) (N, %)	18 (9.7)/9 (4.9)/4 (2.2)	3 (6.0)/3 (6.0)/0 (0)	NS
Cholangitis (N, %)	6 (3.2)	1 (2.0)	NS
(Mild/Medium/Severe) (N, %)	3 (1.6)/3 (1.6)/0 (0)	0 (0)/1 (2.0)/0 (0)	NS
Bleeding (N, %)	2 (1.1)	2 (4.0)	NS
(Mild/Medium/Severe) (N, %)	0 (0)/1 (0.5)/1 (0.5)	0 (0)/1 (2.0)/1 (2.0)	NS
			(Fisher's exact test)

NS: not significant

Several factors related to complications during or after the endoscopic procedure in Group B and in all the cases were examined. In Group B, only EST significantly affected the occurrence of complications after the endoscopic procedure (p = 0.04). Meanwhile, in the all cases, female sex (p = 0.02) and long diameter of CBD (p = 0.04) were the factors related to complications during the endoscopic procedure. Furthermore, antithrombotic drug use (p = 0.04) and pancreatic stent placement (p = 0.04) were the factors related to the complications after the endoscopic procedure. PS, concomitant diseases (dementia, cerebrovascular disorder), periampullary diverticulum, reconstructed gastrointestinal tract, procedure time, dose of MDZ, diameter of stones, number of stones, physician, physician exchange, EPBD and EPLBD were non-significant factors during or after the endoscopic procedures in Group B and in all the cases.

No significant differences were observed between Group A and Group B with regards to length (15.2±1.2 vs. 17.6±1.5 days) or cost of hospitalization (\867,900 vs. \941,160). The cholecystectomy rate after the endoscopic procedures was higher in Group A than in Group B (58.4% [108/185] vs. 24.0% [12/50], p<0.001); However, recurrence of CBD stones during follow-up period and the mortality rates were not significantly different between the two groups (Table 3).

**Table 3:** Clinical course after the endoscopic procedures.

	Group A (< 85y)	Group B (≧ 85y)	p value
	(n = 185)	(n = 50)	
Length of hospitalization (mean±SD) (days)	15.2±1.2	17.6±1.5	NS
Cost of hospitalization (mean±SD) (\)	867,900	941,160	NS
Mortality (N, %)	2 (1.1)	0 (0)	NS
Cholecystectomy after endoscopic procedures (N, %)	108 (58.4)	12 (24.0)	<0.001
Recurrence of CBD stones during follow-up (N, %)	24 (13.0)	10 (20.0)	NS
Observational period (mean±SD) (years)	4.4±0.9	3.4±0.7	NS
			(Fisher's exact test, Student's t-test)

SD: standard deviation, NS: not significant, CBD: common bile duct

In the univariate analysis, the factors related to non-recurrence were age younger than 85 years (hazard ratio [HR] = 0.48, 95% confidence interval [CI): 0.24–0.98, p = 0.047), no cerebrovascular disorder (HR = 0.54, 95% CI: 0.28–0.94, p = 0.032), and no gallbladder in situ (HR = 0.58, 95% CI: 0.34–0.92, p = 0.042). However, these were not significant in the multivariate analysis (Table 4).

**Table 4:** Factors related to non-recurrence in the univariate or multivariate analysis.

Univariate analysis	HR	95%CI	p value
Age of 85 years or younger	0.48	0.24–0.98	0.047
No cardiac disorder	0.54	0.31–1.06	NS
No cerebrovascular disorder	0.5	0.28–0.94	0.032
No dementia	0.91	0.64–1.32	NS
No anti-thrombotic drug use	0.98	0.60–1.40	NS
No ursodeoxycholic acid use	1.08	0.72–1.40	NS
No periampullary diverticulum	0.8	0.46–1.32	NS
No reconstructed gastrointestinal tract	0.78	0.42–1.26	NS
No cholangitis at diagnosis	1.06	0.64–1.46	NS
No gallbladder in situ	0.58	0.34–0.92	0.042
Multivariate analysis	HR	95%CI	p value
Age of 85 years or younger	0.79	0.35–1.80	NS
No cerebrovascular disorder	0.59	0.29–1.21	NS
No gallbladder in situ	0.68	0.32–1.44	NS
			(Fisher's exact test)

HR: hazard ratio, CI: confidence interval, NS: not significant

## Discussion

This study demonstrated the efficacy and safety of endoscopic treatment for CBD stones in patients aged 85 years or older. Clinical characteristics of these patients included a high prevalence of dementia and cerebrovascular disorders and large CBD diameters and stones, in comparison with those in patients younger than 85 years. Although the complete CBD stone removal rate was slightly higher in Group A, we concluded that endoscopic procedures for CBD stones in patients 85 years or older could be performed safely and effectively because we found no significant difference between Group A and B with regard to recurrence, complication, and mortality rates. Furthermore, the length and cost of hospitalization were not significantly different between the two groups.

As noted earlier, if the elderly are defined as people 85 years or older, data on the effectiveness and safety of ERCP for CBD stones would be limited. Ito et al. [7] reported the efficacy and safety data of patients who underwent EPBD, and defined the elderly as those aged 85 years or older. Thus, the present study is the first to evaluate actual clinical data and outcomes for EST, EPBD, and EPLBD.

Many elderly patients with CBD stones were women with poor performance status, in addition to dementia, cerebrovascular disease, or heart disease. They tended to have large diameter CBD stones, and required low sedation doses. The rates of complete stone clearance in the elderly were reportedly lower, at 71–97%, than that in younger patients [5–7, 10–12]. These results are consistent with the findings of this study (Table 1). The use of antithrombotics and ursodeoxycholic acid and the number of patients with a periampullary diverticulum or reconstructed gastrointestinal tract might increase with age. Although the use of antithrombotics was related to the complication after the ERCP procedure in the all cases, in other cases, this was were not a significant factor related to the complications, unlike the findings in previous studies [5–7,10–12]. Furthermore, we found no significant factor related to the complications during or after the ERCP procedure except when EST was performed in Group B. Despite the small number of patients in Group B, the group had fewer factors related to the complications in the patients aged 85 years or older compared to all patients. Thus, we consider that we could safely perform ERCP procedures for patients aged 85 years or older.

No significant differences in the frequency of complications after endoscopic procedures, including pancreatitis and cholangitis, were observed between the two groups. The frequency of complications such as decreased blood pressure or peripheral oxygen saturation during endoscopic procedures was also not significantly different. These findings were similar to those in previous reports [5,6,10–12]. On the other hand, Ito et al. [7] reported that the prevalence of post-ERCP pancreatitis (PEP) after EPBD was significantly higher in patients younger than 85 years than in patients aged 85 years or older. In general, PEP tends to occur in patients who undergo EPBD [13]. Another study reported that PEP was more likely to occur in younger people than in the elderly [14]. However, the prevalence of PEP was not significantly different between the two groups in this study, which assessed all treatments, including EST, EPBD, and EPLBD.

In this study, we found no significant difference in the recurrence rate of CBD stones between the two groups. However, the incidence of the recurrence of CBD stones tended to be higher in Group B than in Group A. According to former reports, several factors affect the bile duct stone recurrence after stone extraction including no history of cholecystectomy [15]. In this study, the surgical rate was significantly lower in Group B than in Group A. This may have influenced the incidence of the recurrence of CBD stones between the two groups.

This study also focused on both medical costs for CBD stone treatment and hospitalization in elderly patients, which no previous study has addressed. No significant differences in costs and hospitalization were observed between the two groups.

We also considered whether less experienced endoscopists under expert guidance could safely perform endoscopic treatment in patients aged 85 years or older. No significant differences were found between the two groups in terms of endoscopic success rate and incidence of complications associated with the procedures. This suggests that with appropriate expert guidance, even trainees can perform endoscopic treatment in elderly patients with CBD stones. No prior reports have described operator-related differences in management of CBD stones in the elderly.

The limitations of this study included the fact that this was a single-center retrospective study with a small sample size. Second, the division into two groups by age was arbitrary. Third, selection bias in choosing a trainee operator was possible, even though the procedure was supervised by an expert physician.

## Conclusions

Endoscopic treatment for CBD stones in patients aged 85 years or older can be safely performed without increasing medical costs. However, elderly patients still require special consideration during endoscopic treatment.
